# Supplementary material for: Spectral inference reveals principal cone-integration rules of the zebrafish inner retina
Source: Curr Biol. 2021 Dec 6;31(23):5214–5226.e4. doi: 10.1016/j.cub.2021.09.047 (PMC8669161; doi:10.1016/j.cub.2021.09.047)
Supplement: Document S1. Figures S1–S3 [file mmc1.pdf]

Current Biology, Volume *31*

Supplemental Information

Spectral inference reveals principal cone-integration rules of the zebrafish inner retina

Philipp Bartel, Takeshi Yoshimatsu, Filip K. Janiak, and Tom Baden

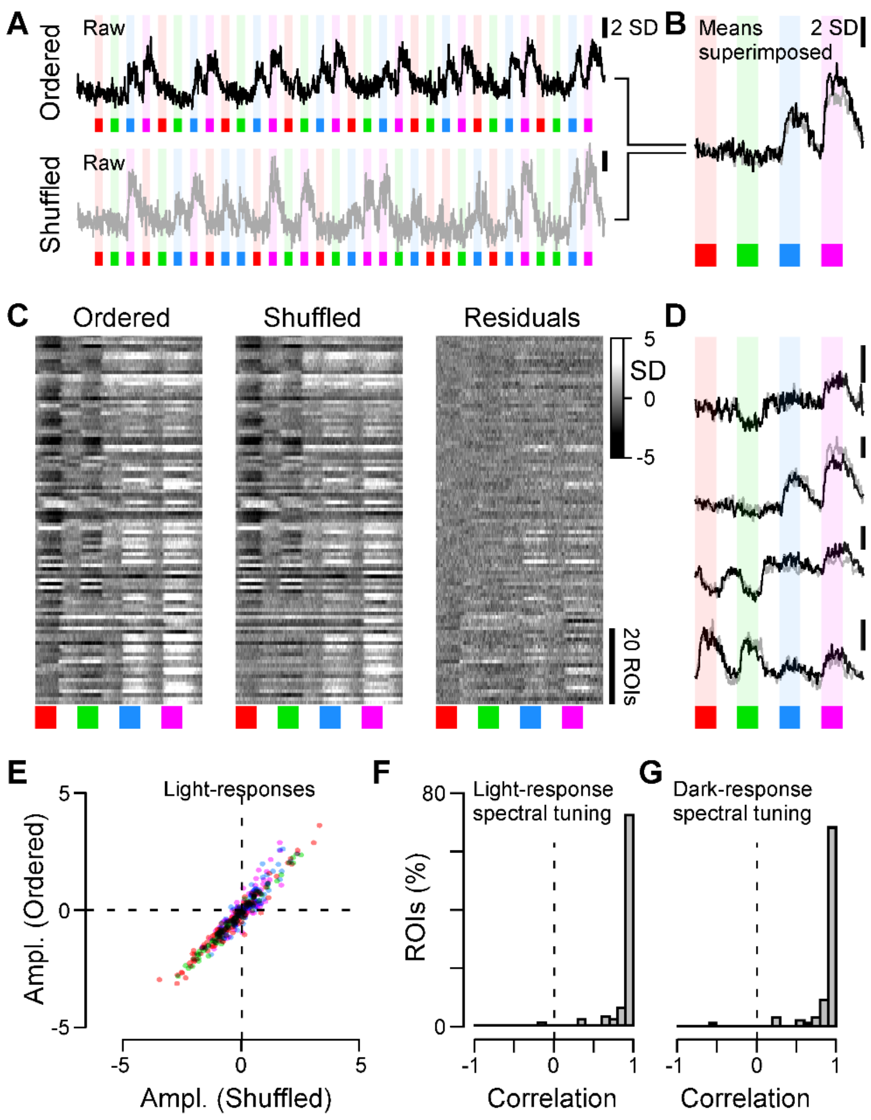

Figure S1 | Comparing responses to ordered versus pseudorandom stimulus sequences. Related to Figure 1. A,B, Example recording from a single ROI responding to four coloured flashes of light (592, 464, 427, 381 nm; 1.5 s, with 1.5 s gaps) presented either in spectral sequence (A, top, black trace, aka. “ordered”) or in pseudorandom sequence (A, bottom, grey trace, aka. “shuffled”), and their stimulus-aligned averages superimposed (B). Note that to facilitate direct comparison, the average flash responses to the pseudorandomised stimulus were concatenated in spectral sequence to resemble the means of the ordered presentation. **C**, Heatmaps of mean responses as in (B) from 100 ROIs (n = 2 fish, n = 3 scan fields) that passed the same quality criterion used for the more spectrally resolved dataset (Methods). Shown are all mean responses to the ordered sequence (left), to the shuffled sequence (middle), and their residuals after subtracting the shuffled from the ordered means (right). **D**, Further examples of means from ordered (black) and shuffled (grey) presentations. Note that generally, responses to both approaches were similar. **E**, Direct comparison of each ROI’s mean response amplitudes during each flash for the ordered versus shuffled condition. Flash-wavelength indicated by the four colour shadings. **F,G**, Quantification of possible differences in spectral tuning functions obtained by either method. Shown are histograms of the linear correlation coefficients between spectral tunings functions based on each ROI’s “light-responses” (F, based on E; correlation mean±SD: 0.84±0.33) and between “dark-responses” (G, i.e. based on four response amplitudes between flashes, correlation mean±SD: 0.81±0.36).

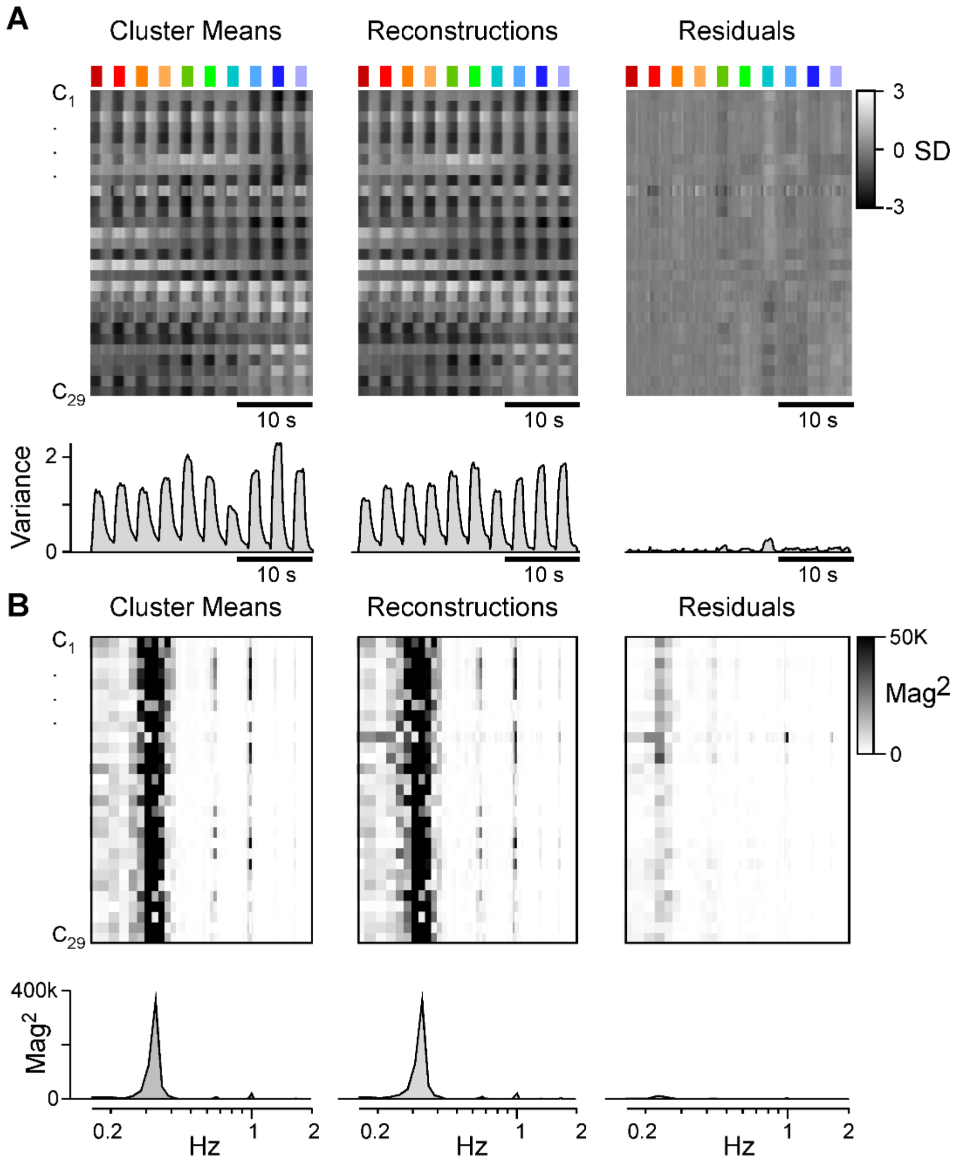

Figure S2 | Cluster reconstruction details. Related to Figure 3. A, Time-aligned heatmaps of all cluster means (left) are shown alongside their corresponding reconstructions (middle) and residuals (right). The time trace below each cluster shows the total variance across all clusters per time point (Methods). **B**, as A, but for magnitude-squared Fourier transforms of each cluster, reconstruction, and residuals. The traces below each panel show the averages of these transforms across all clusters (Methods). Note that for both (A) and (B), residuals retain only a small fraction of the original signal, indicating high reconstruction fidelity. Reconstruction quality of each individual cluster can further be assessed in Appendix 1.

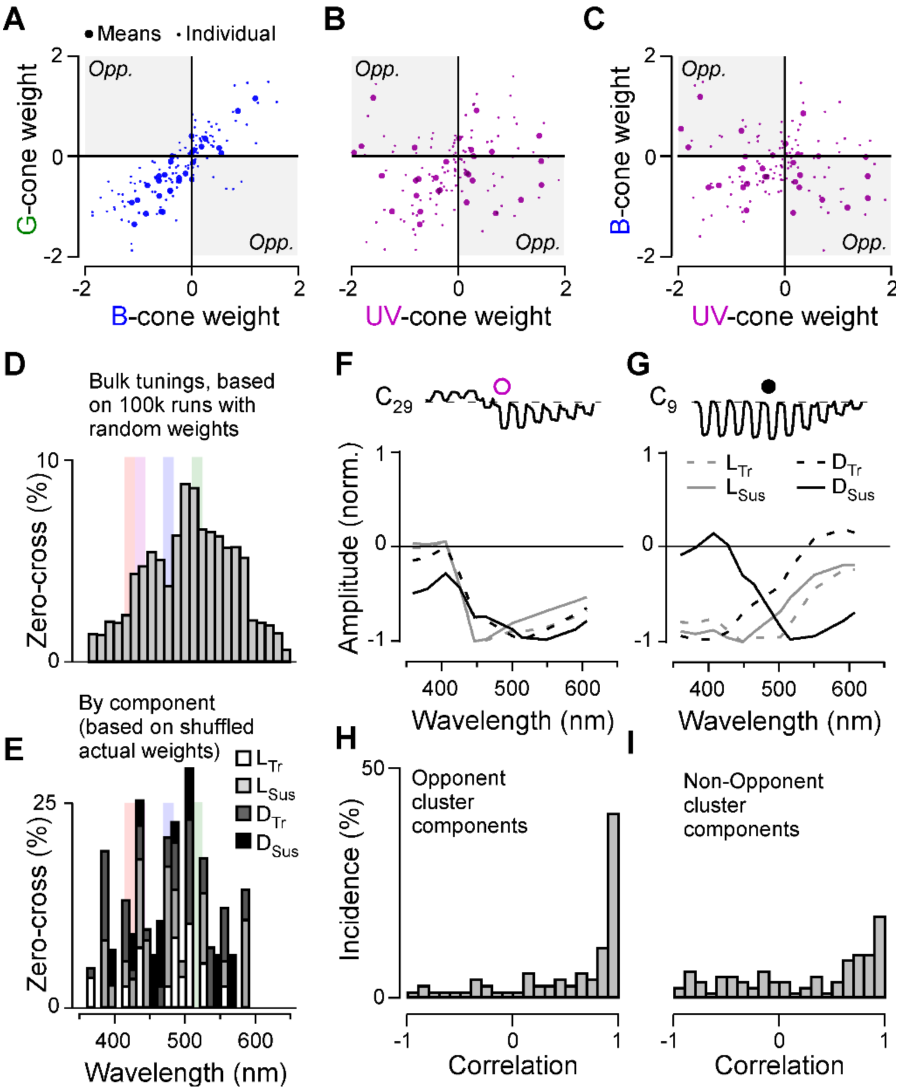

Figure S3 | Spectral tunings and temporal components. Related to Figure 5. A-C, As Figure 5C-E, but showing weight correspondences between green-blue, green-UV and blue-UV cones, respectively. **D**, As Figure 5M, but following based on 100,000 iterations using randomised values (between -5 and 5) for each of the 16 weight variables. **E**, as Figure 5N, but following random permutation of time-components across cones. **F,G**, Spectral tuning functions for two example clusters (C_{29} and C_9, respectively), computed individually by temporal components as indicated. Note that for C_{29} (F), the four tuning functions were similar to each other, while for C_9, the tuning of the dark-sustained component deviated strongly from that of the remaining three components. Corresponding time-component resolved tuning functions are detailed for each cluster in Appendix 1. **H,I**, Distribution of correlations between each cluster's "time-component spectral tuning functions" as illustrated in (F,G), for spectrally opponent clusters (H), and for non-opponent clusters (I).
