# Supplementary figures and images for: Spectral inference reveals principal cone-integration rules of the zebrafish inner retina
Source: Curr Biol. 2021 Dec 6;31(23):5214–5226.e4. doi: 10.1016/j.cub.2021.09.047 (PMC8669161; doi:10.1016/j.cub.2021.09.047)

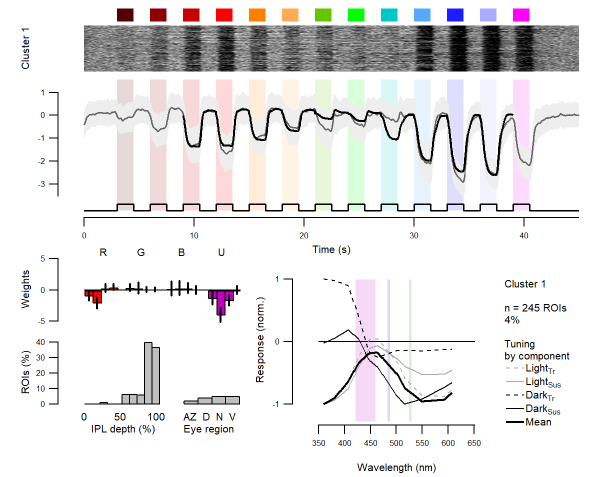

Supplement: Data S1. Detailed summary for each cluster, related to Figure 4 — For each of the 29 clusters as indicated (1 cluster per page), overview of key response aspects and analysis as shown across the main figures. Upper row, from top: Stimulus sequence, heatmap of the response-mean of all ROIs assigned to the cluster (as in Figure 2A), cluster mean ± SD (gray, as Figure 2B) and reconstruction (black, as Figure 4A). Lower row, from top/left: Allocated cone-weights (as Figure 4B), here with SD error bars across each individual ROI that contributes to a cluster, and bottom left: distribution of ROIs across the IPL and eye (as Figures 2C and 2D). Bottom right: Spectral tuning functions extracted from cone weights for each temporal component as indicated (thin lines, as Figures S3F and S3G) and bulk tuning function based on the combination of all temporal components (thick line, as Figures 5F–5K). [file mmc2.zip › curbio_17893_mmc2.tif]
